# Scalable volumetric imaging for ultrahigh-speed brain mapping at synaptic resolution

**DOI:** 10.1093/nsr/nwz053

**Published:** 2019-04-24

**Authors:** Hao Wang, Qingyuan Zhu, Lufeng Ding, Yan Shen, Chao-Yu Yang, Fang Xu, Chang Shu, Yujie Guo, Zhiwei Xiong, Qinghong Shan, Fan Jia, Peng Su, Qian-Ru Yang, Bing Li, Yuxiao Cheng, Xiaobin He, Xi Chen, Feng Wu, Jiang-Ning Zhou, Fuqiang Xu, Hua Han, Pak-Ming Lau, Guo-Qiang Bi

**Affiliations:** 1 Hefei National Laboratory for Physical Sciences at the Microscale, and School of Life Sciences, University of Science and Technology of China, Hefei 230027, China; 2 CAS Key Laboratory of Brain Function and Disease, and School of Life Sciences, University of Science and Technology of China, Hefei 230027, China; 3 Institute of Automation, Chinese Academy of Sciences, Beijing 100190, China; 4 University of Chinese Academy of Sciences, Beijing 100049, China; 5 School of Information Science and Technology, University of Science and Technology of China, Hefei 230027, China; 6 National Engineering Laboratory for Brain-inspired Intelligence Technology and Application, University of Science and Technology of China, Hefei 230027, China; 7 Wuhan Institute of Physics and Mathematics, Chinese Academy of Sciences, Wuhan 430071, China; 8 CAS Center for Excellence in Brain Science and Intelligence Technology, Shanghai 200031, China

**Keywords:** fluorescence microscopy, brain mapping, tissue clearing, immunostaining, activity trace mapping

## Abstract

The speed of high-resolution optical imaging has been a rate-limiting factor for meso-scale mapping of brain structures and functional circuits, which is of fundamental importance for neuroscience research. Here, we describe a new microscopy method of Volumetric Imaging with Synchronized on-the-fly-scan and Readout (VISoR) for high-throughput, high-quality brain mapping. Combining synchronized scanning beam illumination and oblique imaging over cleared tissue sections in smooth motion, the VISoR system effectively eliminates motion blur to obtain undistorted images. By continuously imaging moving samples without stopping, the system achieves high-speed 3D image acquisition of an entire mouse brain within 1.5 hours, at a resolution capable of visualizing synaptic spines. A pipeline is developed for sample preparation, imaging, 3D image reconstruction and quantification. Our approach is compatible with immunofluorescence methods, enabling flexible cell-type specific brain mapping and is readily scalable for large biological samples such as primate brains. Using this system, we examined behaviorally relevant whole-brain neuronal activation in 16 c-Fos-shEGFP mice under resting or forced swimming conditions. Our results indicate the involvement of multiple subcortical areas in stress response. Intriguingly, neuronal activation in these areas exhibits striking individual variability among different animals, suggesting the necessity of sufficient cohort size for such studies.

## INTRODUCTION

Large-scale 3D imaging has become an increasingly important approach in biology, especially for the study of brain circuits [1]. Systematic brain-mapping efforts are building the foundations for our understanding of brain architecture [2–4]. Meanwhile, targeted labeling and brain-wide tracing together with specific manipulations are revealing circuitry mechanisms underlying brain functions [5–8]. The rapid expansion in both fronts demands efficient imaging methods. Traditionally, 3D information of fixed brains and other biological samples was obtained by reconstructing series of 2D images of mechanically cut thin sections. Using such samples with fluorescent protein expression or immunofluorescence staining, epifluorescence or confocal microscopy can resolve fine structures such as neuronal synapses [9]. However, the low throughput of this approach makes whole-brain data reconstruction and analysis a tedious task. Several techniques with automated serial sectioning and imaging methods were developed to overcome this problem [9–16]. Recent advances along this line have allowed for improving the imaging speed to ~3 days per mouse brain at synaptic resolution [14] or ~2.4 hours per mouse brain but at a reduced resolution [15]. Still, it is highly desirable to be able to obtain high-resolution images at very high speed for high-volume tasks such as whole-brain mapping of many animals or of large primates.

The past decade has also seen various tissue-clearing techniques that greatly improve the transparency of brain samples [17]. Combined with these techniques, serial light-sheet microscopy enables fast but low-resolution volumetric imaging of cleared entire brains without mechanical sectioning [8,18,19]. At cellular resolution, such an approach can achieve imaging of the whole mouse brain within 2 hours [8]. Light-field microscopy can also be used for fast imaging of cleared brains at cellular resolution [20]. Importantly, several clearing methods are compatible with immunofluorescence staining, allowing for imaging structures not labeled by genetically encoded fluorescent proteins [21,22], even though such staining usually requires prolonged time to complete because of the limited diffusion rate of antibodies.

Here, we report a new, scalable strategy implementing high-speed Volumetric Imaging with Synchronized on-the-fly-scan and Readout (VISoR) for large samples at high resolution. For small samples such as cultured cells [23] and *Drosophila* larvae [24], state-of-the-art light-sheet microscopy methods have already achieved high-resolution and camera-limited imaging speed. However, these methods cannot be used for larger samples because they rely on fast movement of the imaging section across the sample along the axial direction; the range of such movement is thus limited by the working distance of the imaging objective. Our solution is to move the sample horizontally under the V-shape objectives over nearly unlimited range, while using a single-pass beam scan to eliminate motion blur, thus allowing fast imaging of large samples without compromising image quality. As a proof of principle, we designed and built a system implementing VISoR that can complete the image acquisition of an entire adult mouse brain within 1.5 hours at 0.5 × 0.5 × 3.5 µm^3^ voxel size, with individual synaptic spines in cortical neurons readily visible. We have also developed an optimized pipeline for thick brain-slice sample preparation as well as programs for semi-automated 3D reconstruction and analysis. The procedure is readily compatible with antibody staining that can be completed within a few days, thus enabling versatile high-throughput cell-type-specific 3D brain mapping.

## RESULTS

**Figure 1:**
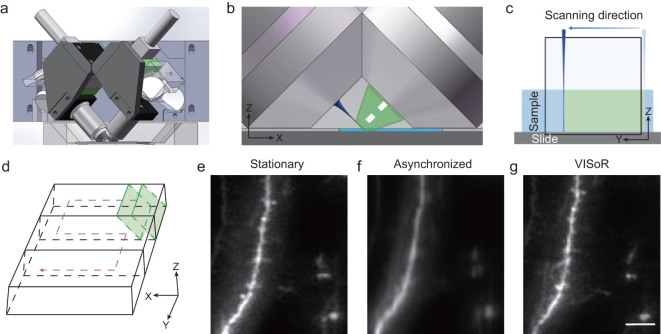
The mechanism and implementation of VISoR. (a) The core optics of the system consists of two perpendicular objective lenses mounted 45 degrees oblique to the sample surface. (b) Enlarged view of the working area, showing the geometric configuration of the two objectives over the sample slice (light blue), along with the illumination beam (dark blue) and fluorescence collection path (green). (c) The imageable area in the object plane (in conjugation with the camera sensor) is illuminated by the scanning light beam synchronized to the camera readout. Note that only half of the sensor area is utilized for the acquisition of single-channel images. (d) Volumetric imaging is implemented by synchronous single-pass beam scan and on-the-fly readout over the surface of a sample slice with a zigzagged motion path. (e)–(g) Fluorescence images of the same sample area obtained with stationary imaging (e), non-synchronized on-the-fly imaging in ‘light-sheet’ mode (f) and VISoR imaging (g). Scale bar: 10 µm.

The basic configuration of our system consists of an illumination objective through which a scanning light beam enters a thick-slice sample at a 45-degree angle to the sample surface and an imaging objective positioned perpendicular to the illumination plane (Fig. 1a and b), similar to the V-shaped light-sheet microscope typically used for cellular imaging [25]. Both objectives are immersed in refractive-index-matched solution (see the ‘Methods and Materials’ section) so that high-quality images of oblique sample sections can be obtained at the camera sensor (Supplementary Fig. 1). The geometry and working distance of the objectives constrain the imageable sample thickness to ~300 µm (Fig. 1b). Using a 20× imaging objective with system magnification reduced to ~13×, the effective imaging area on the object plane (corresponding to half of the camera sensor, ~1000 × 2000 pixels) covers an oblique section of ~420 × 1000 µm (Fig. 1c).

To achieve fast volumetric imaging over a large range, we configured the system so that the sample stage can move smoothly without stopping while images of oblique optical sections are acquired continuously (Fig. 1c and d). This approach has also been independently developed in the recent ‘open-top light-sheet microscopy’ and ‘oblique light-sheet tomography’ [26,27]. However, because the movement is not along the axial direction, the lateral component of motion naturally causes smear (motion blur) of the acquired images that is proportional to the speed of the moving stage (Fig. 1e and f). To overcome this problem, we implemented a thin excitation beam (3–5 µm diameter) to scan through the continuously moving sample, synchronized with the image-acquisition cycle. At the 100 Hz scanning and imaging cycle with the stage moving at 0.5 mm/s, the effective excitation time for each voxel is less than 100 µs, within which the motion is less than 0.05 µm. With this strategy, we eliminate virtually all motion blur and achieve high image quality indistinguishable from stationary imaging, with dendritic spines of cortical neurons clearly visible (Fig. 1e–g). In principle, motion blur can also be reduced using strobe-light illumination in a regular light-sheet configuration. This, however, requires much higher light power to achieve similar illumination. Thus, the synchronized beam scan is optimized in light efficiency.

Our prototype system allows continuous image acquisition from large samples at a volume rate of 0.3 × 1.0 × 1.0 mm^3^ per second and a voxel size of 0.5 × 0.5 × 3.5 µm^3^ with optical resolution of 1 µm lateral and 5 µm axial (Supplementary Fig. 2). This speed is primarily limited by the data rate of the camera (400 million voxels per second). To test the capacity of this new system in whole-brain imaging, we started with fixed brains from *Thy1*-YFP-H mice [28]. An embedded mouse brain was cut into ~50 slices with 300-µm thickness each, to match the imaging depth of the optical system (Fig. 1b). A pipeline was developed to optimize brain fixation, slice cutting and handling, as well as tissue clearing and mounting procedures (Supplementary Fig. 3a). We have also developed a system to automatically detect the edges of all mounted brain slices, so that the imaging system can directly move to the target based on correlated coordinates (Supplementary Fig. 3b). Because of the oblique configuration of excitation and imaging light paths relative to the sample surface, tissue clearing and refractive-index matching are critical, as a mismatched immersion solution could lead to severe aberration (Supplementary Fig. 4). With optimized sample preparation and imaging conditions, high-quality volumetric image series or ‘columns’ can be obtained (Supplementary Video 1). Adjacent image columns were taken with about 10% overlap, in order to facilitate the alignment for reconstruction of the entire slice (Supplementary Videos 2 and 3). In our experiments with 20× objective (13.3× effective magnification), 200-Hz scanning and 1-mm/s stage speed, corresponding to ~0.5 × 0.5 × 3.5 µm^3^ voxel size, volumetric data acquisition of a complete set of about 50 300-µm slices of a whole mouse brain can be completed in less than 1.5 hours.

**Figure 2:**
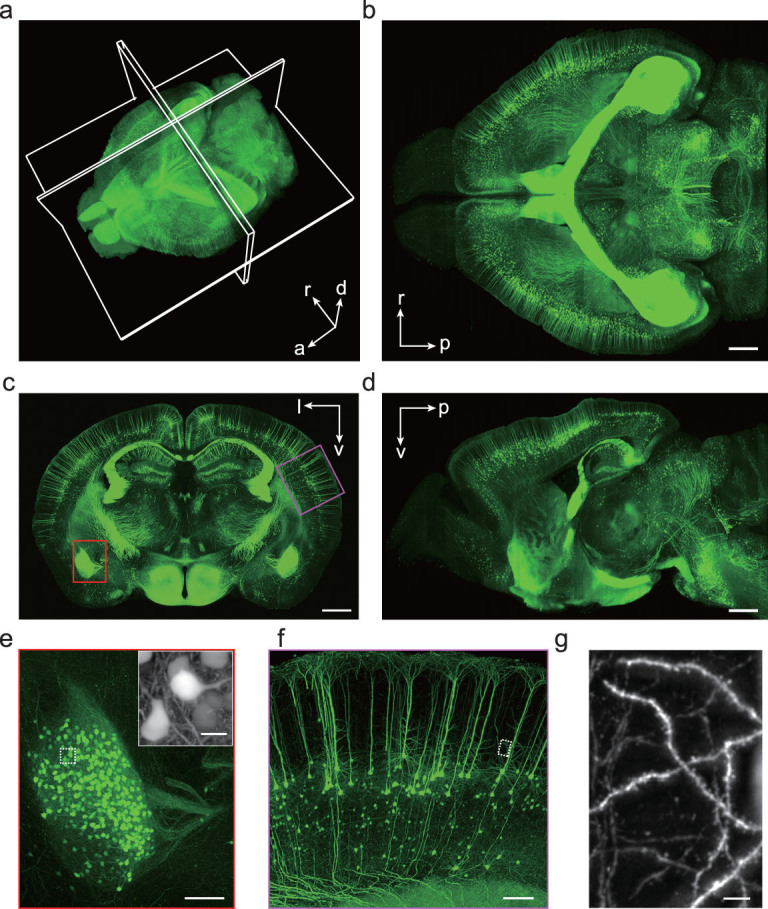
Whole-brain VISoR imaging of a Thy1-YFP transgenic mouse. (a) 3D reconstructed mouse brain. Maximum intensity projections (MIPs) of an 800-µm horizontal section, a 300-µm coronal section and a 150-µm sagittal section are displayed in (b), (c) and (d), respectively. The position of each section is illustrated in (a). l, left; r, right; d, dorsal; v, ventral; a, anterior; p, posterior. (e) Enlarged view of the red box in (c) showing the left amygdala. Inset: an enlarged view of the white rectangle area. (f) Enlarged view of the magenta box in (c) showing part of the cortex. (g) Enlarged view of the white rectangle area in (f). Note that the inset of (e), and (g) are MIPs of the corresponding areas with projection direction perpendicular to the imaging plane, i.e. 45° oblique to the slice surface, thus appearing to be different from the direct magnification of the corresponding rectangle areas. Scale bars: (b), (c), (d), 1000 µm; (e), (f), 200 µm; inset of (e), 20 µm; (g), 10 µm.

Reconstruction of the whole-brain volumetric imaging data was performed with custom software that used minimal morphing to compensate for structural deformations during sample preparation (see the ‘Methods and Materials’ section; Supplementary Fig. 5). Aside from some misalignments between adjacent slices, the reconstructed whole brain shows well-preserved, consistent 3D structural details (Fig. 2a–d and Supplementary Video 4). Neuronal axons and dendrites as well as dendritic spines can be clearly visualized (Fig. 2e–g). From Fig. 2g, we were able to identify spines with an average density of 0.4/µm, similar to that reported previously based on two-photon microscopy [29]. Similar results were obtained from all four *Thy1*-YFP-H mice brains tested. In addition, high-quality data were also acquired for various brain samples labeled using neurotropic viruses, including a Semliki Forest virus (SFV) variant that sparsely labels neurons within 24 hours in the hippocampus (Supplementary Fig. 6) and a vesicular stomatitis virus (VSV) that labels dopaminergic neurons in the ventral tegmental area (VTA) through nerve terminal infection at the nucleus accumbens (NAc) (Supplementary Fig. 7).

**Figure 3:**
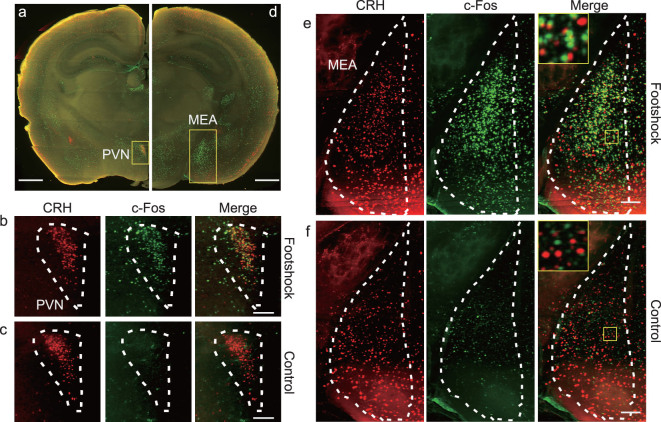
Dual-channel VISoR imaging of c-Fos expression revealing differential stress-elicited activation of CRH neurons in different brain areas. (a) CRH and c-Fos expression in a virtual coronal section (A/P: bregma –0.72~–0.9) of a CRH-ires-Cre^+/+^::Ai14^+/–^ transgenic mouse exposed to foot-shock stimulation. Green, c-Fos immunostaining; red, tdTomato expressed in CRH neurons. (b) Magnified view of the PVN area as shown in (a) from the same animal. (c) Magnified view of the PVN area from a control animal not exposed to foot-shock stimulation. (d) CRH and c-Fos expression in a virtual coronal section (A/P: bregma –1.58~–1.78) contain the MEA from the same animal as in (a). (e) Magnified view of the MEA area as shown in (d). (f) Magnified view of the MEA area from a control animal. MIP of thinner (7 µm) virtual sections are used for (b) and (c) in order to visualize single cells. Scale bars: (a), (d), 100 µm; (b), (c), (e), (f), 100 µm.

An advantage of hydrated sample preparation is its compatibility with immunofluorescent staining. This allows convenient labeling of different cell types or states, enabling cell-type specific brain connectivity and activity mapping. As an example, we used c-Fos antibodies to label activated neurons in the brains of CRH-ires-Cre^+/+^::Ai14^+/–^ transgenic mice with or without brief foot-shock stimuli (see the ‘Methods and Materials’ section) and performed dual-color VISoR imaging and cell counting in different brain areas (Fig. 3). In the paraventricular nucleus (PVN) of hypothalamus where CRH neurons were observed to form a dense cluster (Fig. 3a and b), more than half of these neurons (430 out of 834) in the stimulated animal were c-Fos positive (Fig. 3a and b), whereas very few cells (37 in total) in the control animal were activated, among them most were CRH neurons (35 out of 771 CRH neurons) (Fig. 3c). This is consistent with the role of CRH neurons of the PVN in stress response [30]. Interestingly, in the medial amygdala (MEA) where CRH neurons were more dispersed (Fig. 3d), the CRH-expression and c-Fos labeling exhibited a near orthogonal pattern in the stimulated animal, with 104 CRH neurons being activated out of 1018 total CRH neurons and 569 total activated neurons (Fig. 3d and e). Again, few neurons (63 in total) in MEA were found activated in the control animal (Fig. 3f). Thus, the foot-shock stress activated primarily CRH neurons in the PVN, but primarily non-CRH neurons in the MEA (which do contain many CRH neurons), suggesting that the CRH neurons in the MEA may have different functions from those in the PVN. These results also demonstrate the effectiveness of our system in combining fast 3D fluorescence imaging with antibody staining to investigate the active roles of specific cell types in brain function.

**Figure 4:**
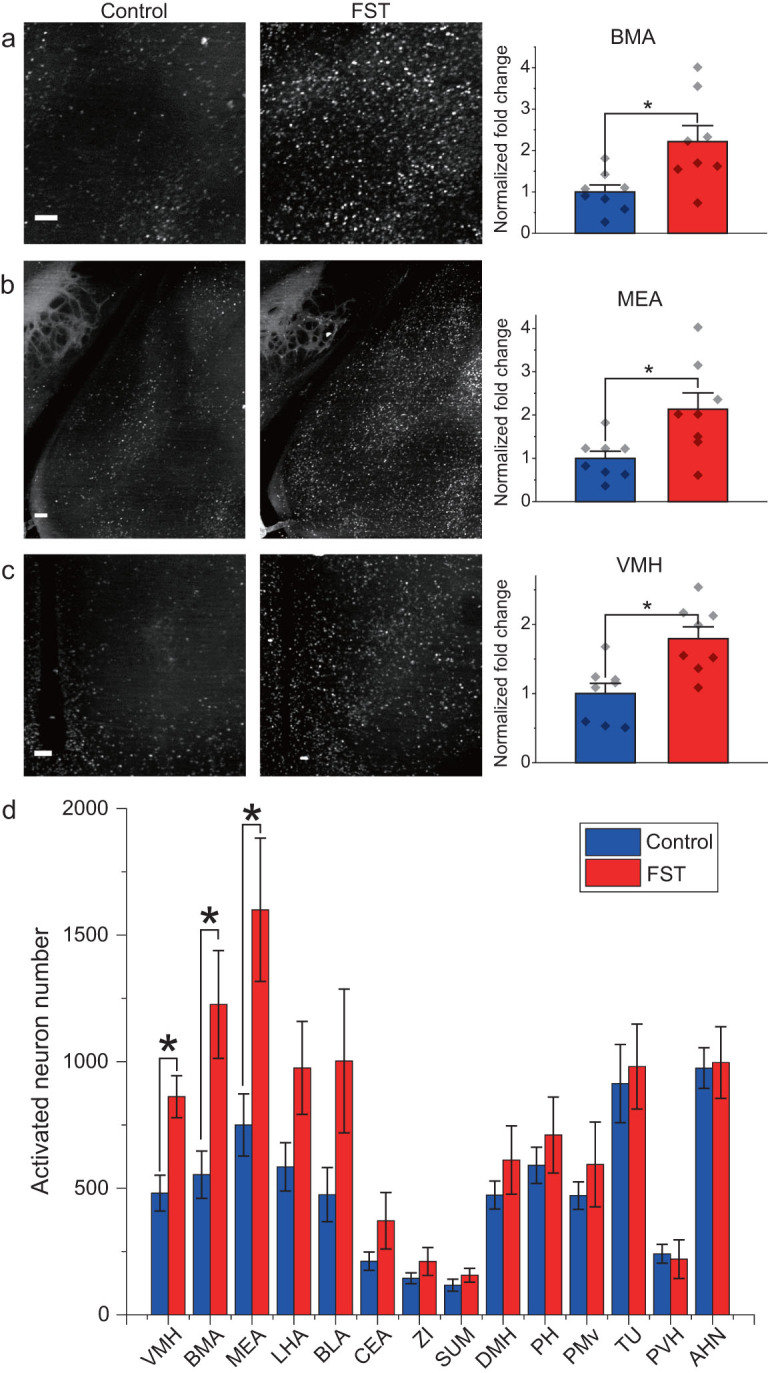
c-Fos activation in mouse brains in response to forced swimming stress. (a)–(c) Example images and the fold changes in the number of c-Fos-expressing neurons in BMA, MEA and VMH of the FST group over the control group, respectively. (d) Analysis of c-Fos activation in multiple subcortical brain areas. For all panels, *n* = 8 per group, ^*^ indicates FDR *q*-value <0.05, based on *P*-values from unpaired two-sample *t*-test comparing the FST group to the control group. Scatter points represent the c-Fos activation of each animal. Error bars, mean ± SEM. Scale bars: (a)–(c), 100 µm.

**Figure 5:**
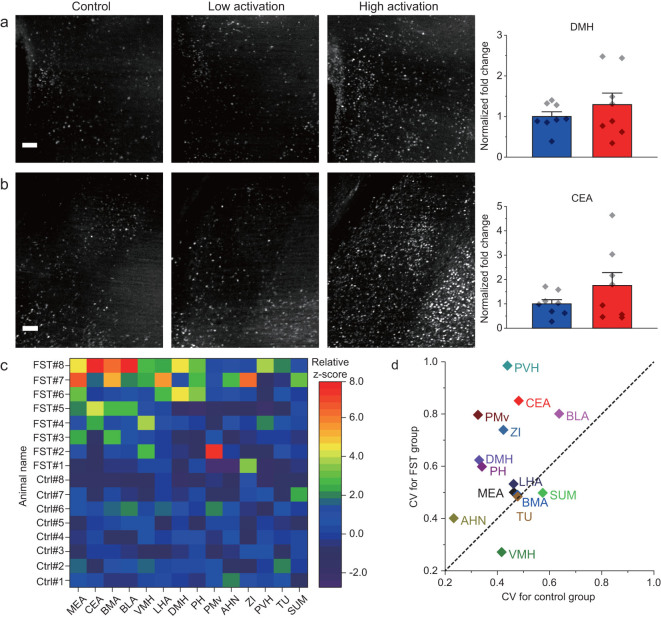
Individual variability of neuronal activation in animals by forced swimming stress. (a) and (b) Summary of c-Fos activation in the DMH and CEA, where substantial individual variability is seen across different animals. (c) Relative z-score plot of 14 brain areas from 16 animals. (d) Coefficient variation plot of 14 brain areas between FST group and control group. *n* = 8 for each group. For (a) and (b), unpaired two-sample *t*-test comparing the FST group to the control group yielded no significant differences. Scatter points represent the c-Fos activation of each animal. Error bars, mean ± SEM. Scale bars: (a) and (b), 100 µm. Abbreviations of brain areas used in (c) and (d) and not mentioned in main text are ventral pre-mammillary nucleus (PMv), anterior hypothalamic nucleus (AHN), zona incerta (ZI), paraventricular hypothalamic nucleus (PVH), tuberal nucleus (TU) and supramammillary nucleus (SUM).

Taking advantage of the high speed of the VISoR system, we investigated neural circuits involved in stress response under forced swimming test (FST). Eight out of 16 c-Fos-shEGFP mice underwent FST, with the other eight animals serving as the control group. To map neuronal activity traces, the animals were sacrificed 1.5 hours after FST (or corresponding resting condition for the control group) for whole-brain VISoR imaging. We observed that, in animals from both groups, many cortical and subcortical areas exhibited substantial neuronal activation as indicated by EGFP expression (Supplementary Video 5). Upon examination of 14 subcortical areas known to be involved in stress response and emotion, several areas, including the basomedial amygdala nucleus (BMA), medial amygdala nucleus (MEA) and ventromedial hypothalamic nucleus (VMH), showed significantly greater neuronal activation in the FST group as compared with the control group (Fig. 4a–c). The population data for many brain areas exhibited marked individual variability within the FST and even the control groups, such as the BMA (Fig. 4a) and the MEA (Fig. 4b), as also reflected by the large error bars in Fig. 4d. This variability was more apparent in some areas such as the dorsomedial nucleus of hypothalamus (DMH) and central amygdala nucleus (CEA), where the difference between the FST and control groups was not significant (Fig. 5a and b).

To have a more systematic view of the individual variabilities in neuronal activation, we plotted the relative z-scores that were computed based on the mean and standard deviation of c-Fos activation in the control group (see the ‘Methods’ section) for each of the 14 subcortical areas from all 16 animals (Fig. 5c). It is obvious that, even for the areas that were ‘consistently’ activated by forced swimming, several FST individuals behaved more like controls. On the other hand, for areas that did not exhibit significant difference across the two groups, such as the CEA, basolateral amygdala nucleus (BLA) and posterior hypothalamic nucleus, several individuals showed strong activation after FST (Fig. 5c). Furthermore, when we plotted out the variability of neuronal activation in these areas as assessed by the coefficient of variation (CV) for the FST versus control groups, the majority of the areas exhibited higher variability upon forced swimming (Fig. 5d), with a few areas exhibiting similar CVs (close to the diagonal line in Fig. 5d). Intriguingly, the VMH showed smaller CV for the FST group than the control group, probably indicating a ‘ceiling effect’ due to very strong activation by forced swimming, resulting in reduction of the variation. Although c-Fos-shEGFP expression is not necessarily correlated with neuronal activity linearly in all brain areas, the variability seen here could still reflect individual variability of circuit activation in these animals. We suspect that different animals might have been in different mental states or have different strategies for coping with the imposed behavioral stress. If this is the case, our results underscore the necessity for a sufficient population size for such studies.

## DISCUSSION

The speed, resolution and scalability of the VISoR system make it a suitable tool for high-throughput 3D mapping of the brain structure and activity trace. With continuous on-the-fly imaging, the speed of the VISoR system is mainly constrained by the data-acquisition bandwidth. For certain brain-mapping applications where synaptic resolution is not critical, a VISoR system can achieve much higher throughput, such as one entire mouse brain within 15 minutes at a pixel resolution of ~2 µm. With the aim of high throughput, we chose a relatively low NA (0.5) objective and used a low magnification for large field of view. The pixel size in the *x*–*y* plane (~0.5 × 0.5 µm), by Nyquist Law, tightly agrees with the theoretical and measured optical resolution of the system (Supplementary Fig. 2). On the other hand, the measured axial resolution [31] of the system is 5 µm, which is limited by the beam width of the illumination. This resolution should be improved if morphological analysis of fine structures, such as spines and single axonal fibers, is desired. For this purpose, the axial resolution may be improved with other illumination strategies such as a 2-photon Bessel beam [32] or with the dual-view configuration as in diSPIM for cellular imaging [23]. It is worth noting that the VISoR technique can be implemented in different ways and can find applications in the study of 3D structures of other biological and pathological specimens beyond brain mapping.

For brain mapping with VISoR imaging, we adopted a sample-preparation strategy with tissue clearing and staining performed after sectioning that considerably reduces preparation time, from weeks required for antibodies staining of an intact mouse brain [21,22] to a couple of days for staining 300-µm slices. This processing caused some mild expansion of the sections. Similarly to that reported previously by other groups [21,33], microstructures like dendrites and spines were preserved following the expansion (Fig. 2g). A drawback of this approach is the inevitable misalignments between sections in whole-brain reconstruction. Most of the misalignments can be corrected, currently with a semi-automated procedure, which is rather time-consuming. Further improvements in sample preparation, optical design and data processing are needed to achieve efficient and precise alignment in order for VISoR to be used for brain-wide tracing of individual axonal fibers—an important application best done by fMOST [34] and optimized seriel two-photon tomography [12]. For other applications not sensitive to misalignments, VISoR is a fast and versatile scheme. In addition, the same sample-preparation procedure can be used for larger specimens such as primate brain or other large organs. Importantly, because the time for clearing and staining reagents to sufficiently diffuse through the sample is largely determined by the section thickness, the much larger specimens will require a similar amount of time to prepare as for mouse brain. Furthermore, the VISoR system can readily use larger sample-translation stages for larger slices, with no practical limitation on the slice area and with imaging time scaling up linearly. Finally, the system can be extended to accommodate multiple rounds of staining and imaging for various protein and nucleic acid targets. Thus, the VISoR approach is ideally suited for the whole-brain mapping of neuronal cell types, connectivity patterns and activity traces at different levels of details, in a wide range of species from rodents to primates.

## METHODS AND MATERIALS

### Optical setup

The microscope system (Supplementary Fig. 1) consists of a light-source module, a scanning light-beam illumination module, an imaging module, a sample chamber for imaging and a three-axis automatic stage for sample positioning. The light-source module combines four compact lasers with wavelengths 405, 488, 561 and 647 nm (Coherent Inc.) and delivers light to the illumination module, which consists of a galvanometer scanning mirror (GVS011, Thorlabs) and relay lens sets. The optical axis of the illumination path and imaging path are perpendicular and 45 degrees off the sample surface (Supplementary Fig. 1). The illumination and imaging modules have identical front ends, which consist of an objective (UMPLFLN20XW, NA0.5, 20× water immersion, Olympus) and a micropositioner, which allows the potential configuration expansion of dual-excitation/dual-imaging. The focal length of the tube lens in the imaging module is 120 mm, which provides an effective magnification of 13.3×. A dual-channel splitter (Optosplit II, Cairn Research, UK) is mounted between the imaging module and the sCMOS camera (ORCA Flash 4.0 v2, Hamamatsu) to provide two imaging channels at the same time. A combination of emission filters (Semrock Brightline 452/45, 520/28, 593/46 and Chroma ET705/72 M) are mounted in the Optosplit II. The imaging chamber carries the sample and the refractive-index-matched immersion solution, and is mounted on the top of a three-axis motorized stage, which consists of two 1D translation stages (MTS50, Thorlabs) for X/Y-translation and a heavy-duty stage for Z-translation. All major parts are listed in an Excel file that is available to download at https://github.com/BilabOptics/VISoR_code/Design/.

### Computers, electronics and control programs

All electronic components of the system including lasers, galvanometer scanner, DAQ board (PCI-6722, National Instruments) and camera are controlled by a workstation (dual Xeon CPUs, 64GB RAM, Dell) equipped with an 8-terabyte SSD RAID0 array and 10-Gigabit optical fiber networks connected to large-volume storage servers (200 TB, Dell) and computation servers (4 nodes, 28 cores and 128 GB RAM for each node). The whole system is controlled by custom C++ code based on MicroManager [35] core. Imaging data are directly acquired into the SSD array, then transferred to the large-volume storage server for further reconstruction and quantitative analysis.

### Virus preparation

The viral-like particles of Semliki forest virus SFV-EGFP were prepared as follows. Briefly, SFV-EGFP replicon was modified based on the SFV replicon followed by *in vitro* transcription. The SFV-EGFP replicon RNA and helper RNA were co-transfected into BHK21 cells to package SFV-EGFP. The detailed procedures for RNA transcription and virus package were reported in previous studies [36–38]. The mutated VSV rVSV-EGFP-NR7A were prepared as described previously [39,40].

### Animal experiments

All animal experiments were carried out following protocols approved by the Institutional Animal Care and Use Committees of the University of Science and Technology of China (USTC) and Wuhan Institute of Physics and Mathematics, the Chinese Academy of Sciences. All mice used in this study were group-housed with 12 hours light/dark cycle (light on at 7 a.m.) with free access of food and water. Eight-week-old male Thy1-YFP-H (Jax: 003782) mice were used for whole-brain structural imaging. A male C57BL/6 mouse of the same age was used for SFV injection. A DAT-ires-Cre [41] (Jax: 000660) male mouse (8 weeks old) was used for AAV-DIO-BFP-T2A-TVA and VSV-EGFP-NR7A injection. Male CRH-ires-Cre^+/–^::Ai14^+/–^ mice (used at 12 weeks old) were generated by crossing male CRH-ires-Cre+/+ (*Crh^tm^*^1^*^(^^cre^^)^^Zjh^*) [42] (Jax: 012704) and female Ai14^+/–^ (Rosa-CAG-LSL-tdTomato-WPRE::deltaNeo) [43] (Jax: 007908) mice. Male cfos-shEGFP/cfos-tTA [44] (Jax: 018306) aged from 8 to 12 weeks were used for the FST experiment.

SFV injection was performed as described in previous studies [38,45]. Briefly, the mouse was anesthetized with chloral hydrate (400 mg/kg) and placed in a stereotaxic apparatus. SFV-EGFP (2.6 × 10^4^ FFU/mL) was injected into the dentate gyrus of the hippocampus. The mouse was sacrificed 24 hours later. For the VSV tracing experiment, the DAT-Cre mouse was kept under anesthesia as described above. Injection coordinates (in mm) used were: VTA, A/P –3.20 from bregma, L/M –0.40, D/V –4.30; NAC, A/P 1.5 from bregma, L/M –1.1, D/V –4.6. 50 nl of 2 × 10^8^ FFU/mL rVSV-EGFP-NR7A(A/RG) was injected into the VTA 2 weeks after 100 nl of 2 × 10^12^ vg/mL rAAV-DIO-BFP-T2A-TVA was injected into NAc. The mouse was sacrificed 5 days after rVSV-EGFP-NR7A(A/RG) was injected. These experiments were conducted in a biosafety level 2 laboratory.

For foot-shock stimulation, a male CRH-ires-Cre^+/–^::Ai14^+/–^ mouse was given 15 trials of electric shocks (2 seconds, 0.7 mA) randomly spaced in 10 minutes in an isolated behavioral test box with video recording, then rested in a home cage for 80 minutes before sacrificing and perfusion.

For FST, each mouse was put into a 5-L glass beaker with 3 L water at 25°C for 5 minutes. Animal behavior was monitored with video recording. After swimming, the animal was brought out of the water and dried with a heater before returning to the home cage for rest. Ninety minutes after swimming, the animal was sacrificed with a transcardial perfusion for brain harvesting as described below.

For brain harvesting, mice were first anesthetized by intraperitoneal injection of 1% pentobarbital sodium (80 mg/kg). A fixation solution containing 4% paraformaldehyde (PFA) with or without 0.5–4% acrylamide hydrogel monomer solution in phosphate buffered saline (PBS) (w/v) was prepared and stored at 4°C before use. Brains were harvested and incubated in the same fixation solution at 4°C for overnight to 2 days.

### Preparation of brain-slice samples

Fixed brains were cut into 300-µm-thick slices using a vibroslicer (Compresstome VF-300, Precisionary Instruments). The brain slices were then transferred into the clearing solution for 12~24 hours at 37°C with gentle shaking. The clearing solution was either 4% SDS solution [21] or 0.5% PBST. After clearing, the slice samples were washed with 0.1% PBST three times before being mounted onto quartz slides.

For antibody staining, cleared slices were loaded into 12-well plates, permeated and blocked in blocking solution (5% BSA (w/v), 0.3% TritonX-100 in PBS) for 1 hour and then incubated with primary antibody (Rabbit-anti-c-Fos, Santa Cruz SC-52, lot: K0112, dilution 1:500) in blocking solution overnight, followed by washing in 0.3% PBST three times for 1 hour each. The secondary antibody (AF488-tagged donkey-anti-rabbit IgG, Jackson Immuno Research Labs 711–545-152, lot: 129588, dilution 1:200) was 5 hours in blocking solution, followed by washing in 0.3% PBST. The plates were kept on a gentle bouncer at room temperature for all the above steps.

### Fluorescence imaging

Samples on imaging slides were immersed in refractive-index-matching solution [33] (80% Iohexol in PBS, Sigma (D2158)) 4 hours before being mounted in the imaging chamber filled with refractive-index-matching solution. Sixteen-bit images were acquired with synchronized beam-scan illumination and camera-frame readout, with the sample stage moving linearly in the X direction (Fig. 1d). The resultant voxel size is ~0.5×0.5× 3.5µm^3^.

### Whole-brain image reconstruction

Image reconstruction and brain registration were performed using custom programs in ImageJ [46], MATLAB (2016b, Mathworks Inc.) or C++. The obliquely acquired images were first stacked into columns according to their real-world coordinates, then the columns were stitched together to form whole slices based on the coordinates and correlations in their overlay regions.

For the reconstruction of Thy1-YFP brains, we first enhanced the contrast of each slice and then reconstructed the whole-brain structure in four steps. (i) The top and bottom surfaces of each brain slice were fitted into flattened planes by linear regression and interpolation. Structures such as ventricles were avoided in the flattening procedure. (ii) Edges and textures were detected in the opposing surfaces of adjacent slices, with correspondences between the two surfaces extracted using SIFT-flow [47]. (iii) To limit the error accumulation and propagation through multiple slides, we adjusted the correspondences under two additional constrains over all slides, based on the assumptions of smoothness and small deformation—that is, the displacements of the neighboring correspondences should be similar and the displacement vector for the correspondences to should as small as possible. (iv) With the extracted and adjusted correspondences, the moving-least-square method [48] is used to warp each slice with minimal distortion in the sizes and shapes of the slices.

### Image visualization

We developed an adaptive tone-mapping strategy for efficient visualization of the global and local structures in an 8-bit display based on the original 16-bit data. The original high-dynamic-range image is decomposed into a base layer and a detail layer through bilateral filtering [49]. The dynamic range of the base layer is compressed by a global operator and the result is then recombined with the uncompressed detail layer. To cope with the extremely high local contrast exhibited by the fluorescent-protein-labeled neurons in the brain image, we propose a progressive tone-mapping strategy that compresses the dynamic range of the base layer step by step, and the parameters used in bilateral filtering are adaptively determined according to the image content. This allows for capturing the details of both high-brightness structures such as neuronal soma or fiber bundles and low-brightness structures like dendritic spines or axon terminals in the same field of view.

### Cell detection, counting and brain registration

Quantitative analyses were performed using custom programs coded in C++ and Python incorporated with the OpenCV 3 library. The soma of tdTomato-expressing neurons or the nuclei of c-Fos positive cells were detected by finding the local maxima of the Difference-of-Gaussian (DoG) in original 2D images, then segmented using a watershed algorithm based on the local threshold. The 3D somas or nuclei were reconstructed by connecting the detected segments of the same cell nucleus from neighboring images. The cell-detecting algorithm consisted of three steps (Supplementary Fig. 8): (i) Finding all local maxima of DoG of 4× downsized original image and dividing them using a watershed algorithm. (2) Extracting the image patches of the surroundings of each local maxima and finding the area of the cell by threshold. The side length of the patches are three times the predefined cell size. The threshold is set to the average of the value of the local maxima and the mean value of the edges of the patch. (iii) Aligning the areas of cells between neighboring images. After detection, the position, volume, eccentricity and intensity of cells are calculated, then compared to predefined ranges to validate the detected cell.

Automatic brain registration and annotation were performed using a procedure similar to that previously reported [22]. The 3D volumes were downscaled to 25 × 25 × 25 µm pixel size, then flattened and stitched to reconstruct the whole brain. Elastix (http://elastix.isi.uu.nl) was used to register the reconstructed brain to the reference autofluorescence brain (http://www.idisco.info). The position of the cells in the whole brain were refined by the transform parameters of the brain registration.

### Data analysis and statistics

The relative z-score (for a brain area in either an FST or control animal) in Fig. 5c is defined as ${z_i} = \frac{{{x_i} - {{\overline{x}}_{ctrl}}}}{{S{D_{ctrl}}}}$ where ${x_i}$ is the number of activated neurons in the particular brain area of the animal, $\ {\overline{x}_{ctrl}}$ is the average number of activated neurons in the same brain area from all control animals and $S{D_{ctrl}}$ is the standard deviation of the number of activated neurons in the same area from all control animals. For statistical significance, *P*-values from Student's *t*-test are used and FDR *q*-values are calculated [50] to control the false discovery rate in multiple comparisons, as described in figure legends.

### Code availability

Codes for data analysis are openly accessible at https://github.com/BilabOptics/VISoR_code/.

### Data-availability statement

Original data are available from the corresponding authors upon reasonable request.

## Supplementary Material

Supp_nwz053
