# Supplementary material for: Scalable volumetric imaging for ultrahigh-speed brain mapping at synaptic resolution
Source: Natl Sci Rev. 2019 Apr 24;6(5):982–92. doi: 10.1093/nsr/nwz053 (PMC8291554; doi:10.1093/nsr/nwz053)
Supplement: Supp_nwz053 [file supp_nwz053.zip › Wang_VISoR_Supplementary_Materials_tracked.docx]

**Supplementary Figure 1.** **Schematic diagram of the optical system implementing VISoR.**


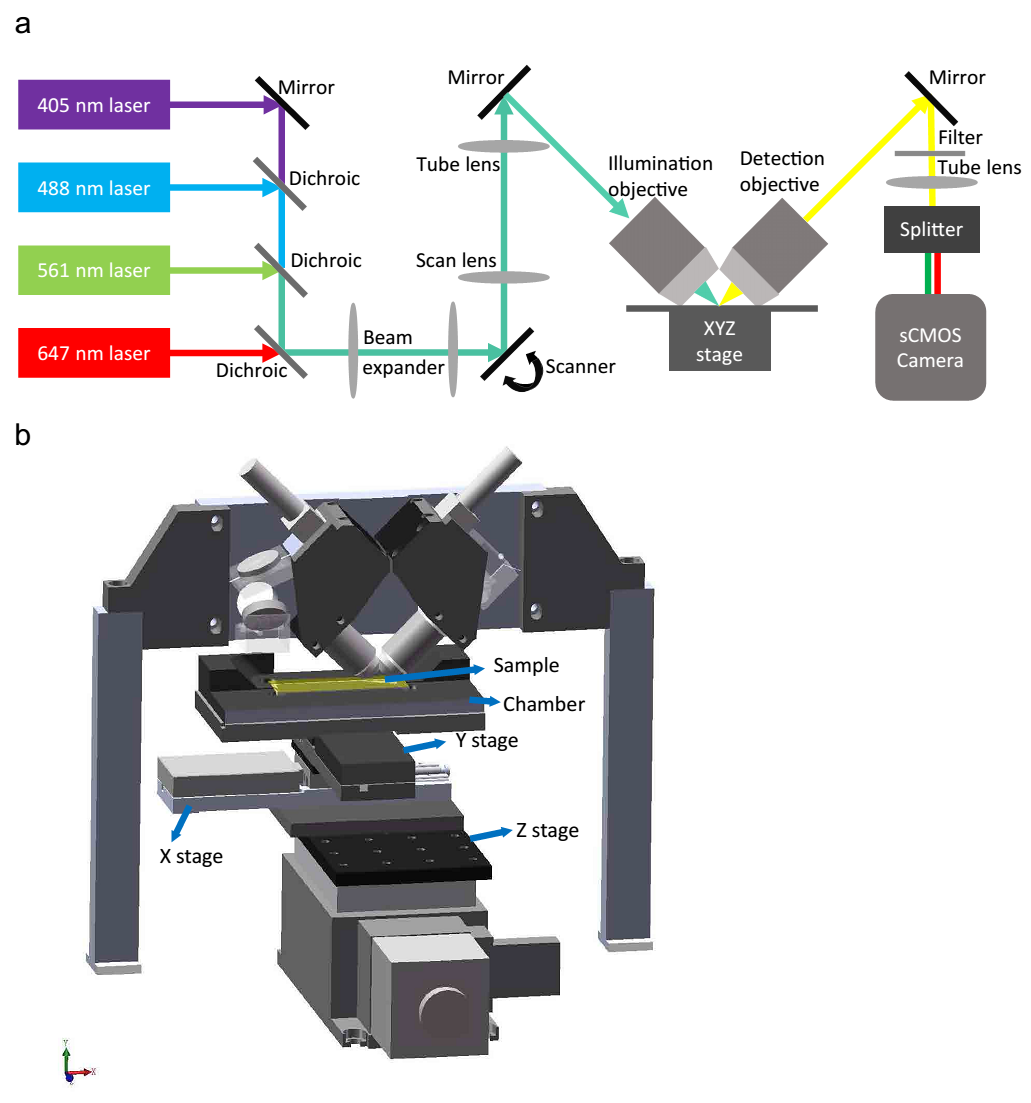


**(a)** The illumination and detection objectives are mounted perpendicular to each other and 45 degree to the surface of the sample. The excitation light beam through the galvanometer scanner and illumination objective forms a scanned light sheet inside the sample that matches exactly the imaging focal plane of the detection objective. The sample chamber mounted on a motorized XYZ stage contains imaging solution with refractive index matched to that of the sample. The detection module consists of emission filters, a tube lens, a dual-channel splitter and a sCMOS camera. Camera readout, laser output and galvanometer scanning are synchronized with an NI DAQ board. **(b)** Optical implementation of the VISoR system.

**Supplementary Figure 2. Optical resolution characterization of VISoR system.**


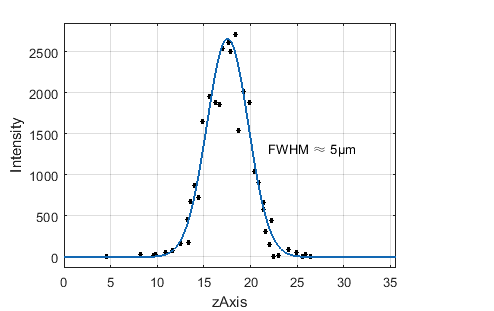

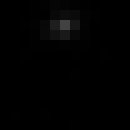

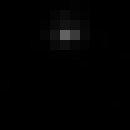

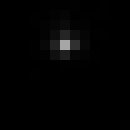

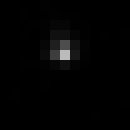

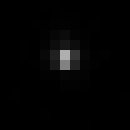

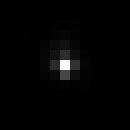

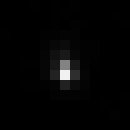

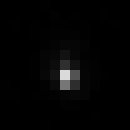

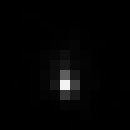

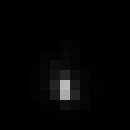

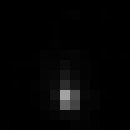

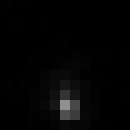

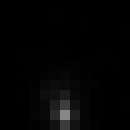

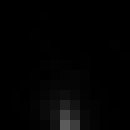

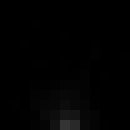


a

b

**(a)** Consecutive images of a 200 nm diameter fluorescent bead acquired at different z planes (distance between two planes is 0.35 μm) show the lateral resolution is around two pixels (0.5 μm per pixel). **(b)** The axial resolution fitted through images in **(a)** is about 5 μm.

**Supplementary Figure 3. Pipeline for sample preparation.**


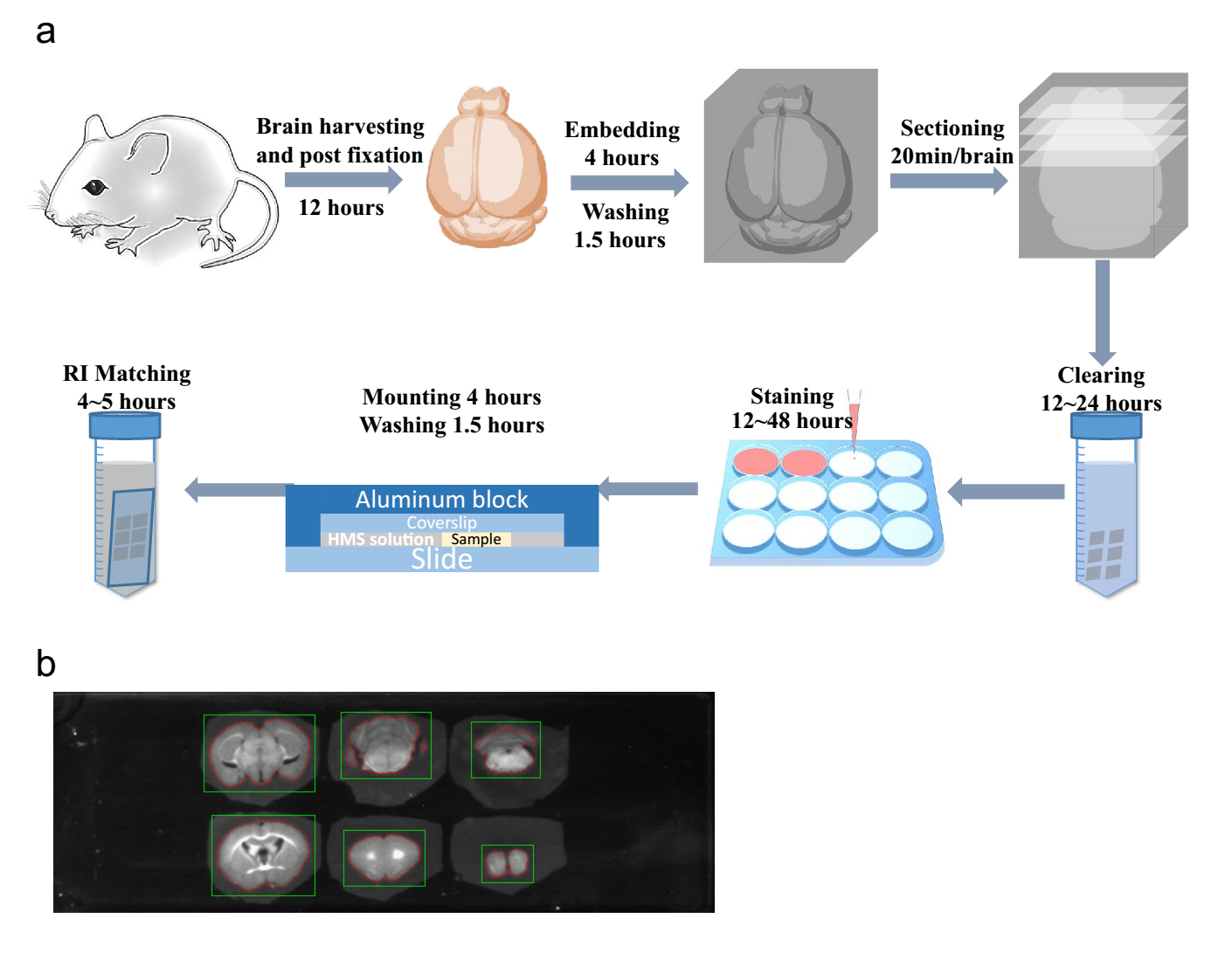


**(a)** The procedure of sample preparation includes following key steps: brain harvesting, embedding, sectioning, clearing, staining (optional), mounting and refractive index matching. With antibody staining, the total time for sample preparation is about 4 days. **(b)** The boundary of each slice (red cycle) is detected by analyzing an pre-acquired image before refractive index matching for further imaging area (green rectangle) determination.

**Supplementary Figure 4. Refractive index matching is essential for high quality imaging.**

**
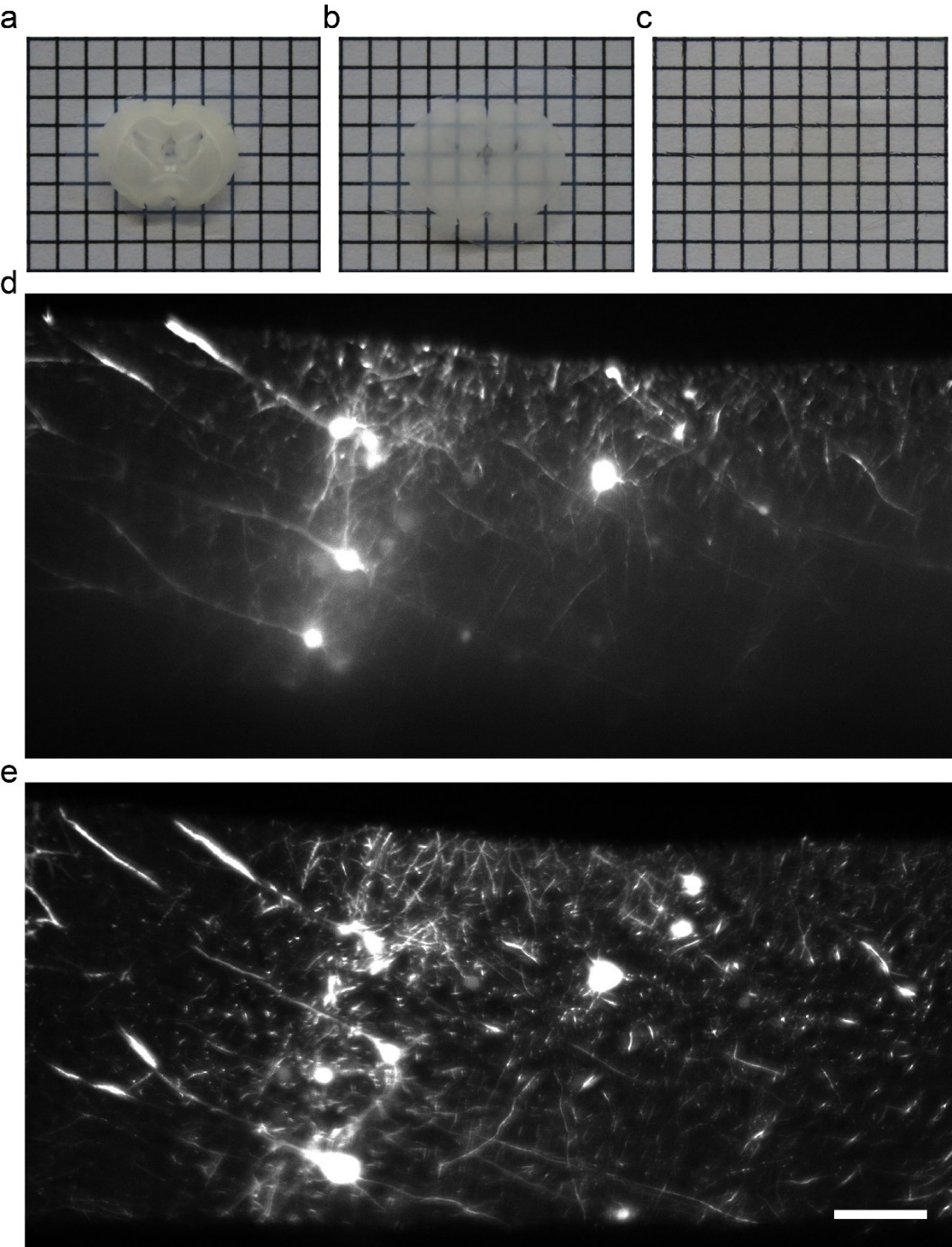
**

**(a-c)** A 300-µm thick brain slice is mostly opaque in PBS **(a)**, becomes translucent after clearing with 4% SDS for 24 hours **(b)**, and fully transparent after refractive index matching in 80% iohexol solution **(c)**. **(d)** An example VISoR image of a cleared brain slice in PBS shows strong scattering and aberration especially at the lower part of the slice. **(e)** A nearby section of the same sample after refractive index matching, taken under otherwise the same imaging conditions as in **(d)**, showing considerably reduced scattering and aberration. Scale bars: **(a)**, **(b)**, **(c)**, 2 mm per grid; **(d)**, **(e)**, 100 µm.

**Supplementary Figure 5. Whole brain data reconstruction workflow for VISoR imaging data.**

**
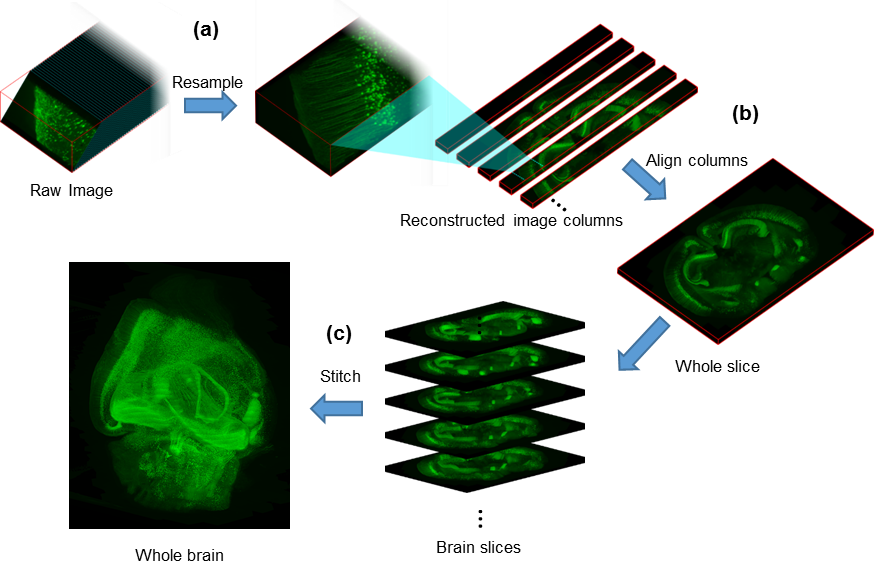
**

**(a)** The raw oblique image sequence is resampled into normal xyz coordinates by each column. **(b)** The reconstructed image columns of a single slice are aligned into an intact slice. **(c)** All slices of a brain are stitched together sequentially.

**Supplementary Figure 6. Hippocampal neurons sparsely labeled with Semliki Forest virus (SFV).**


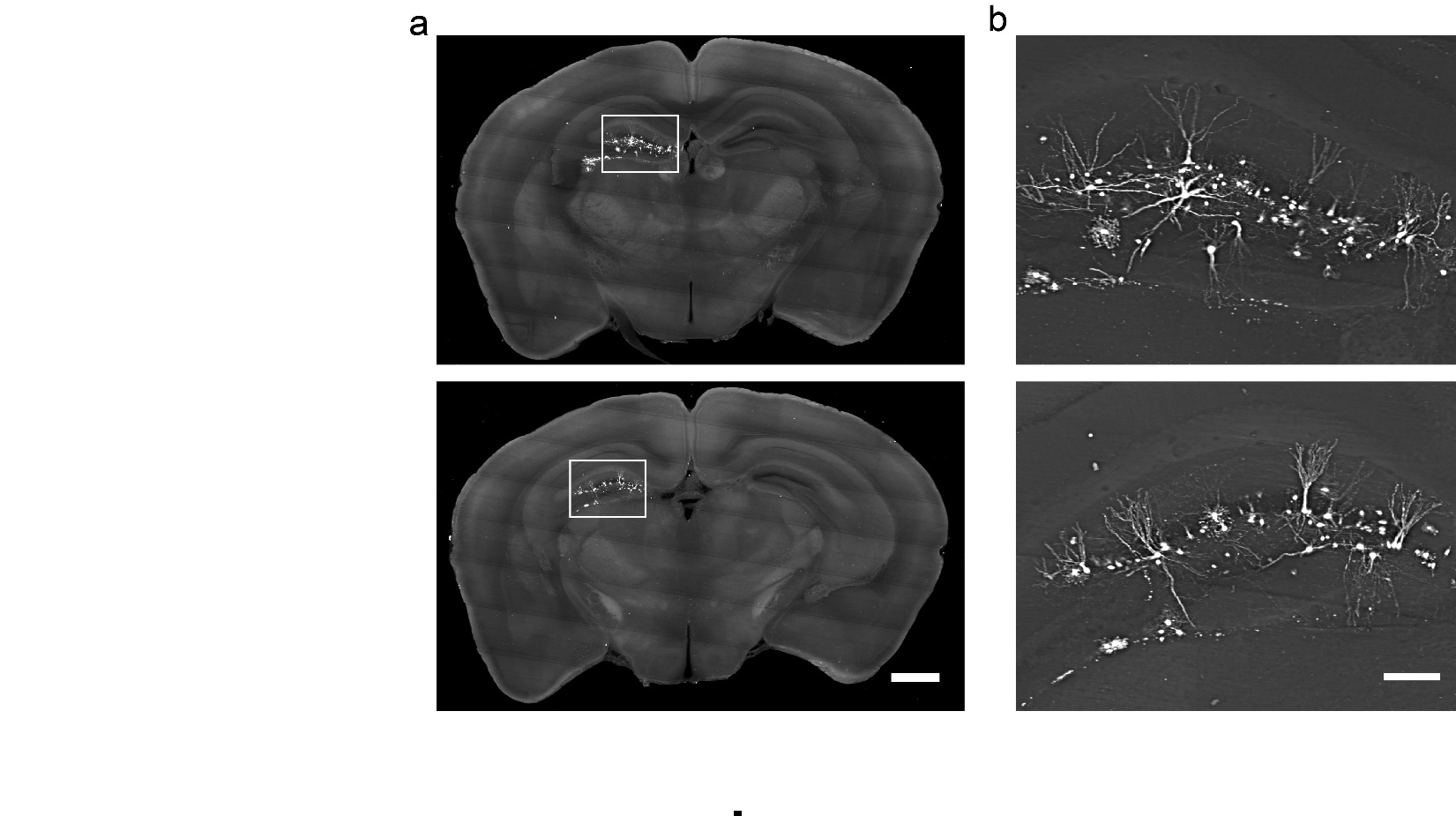


(**a)** Brain sections showing sparsely labeled cells in the hippocampus 24 hours after SFV-EGFP injection. **(b)** Magnified view showing diverse neuronal morphology. Scale bars: **(a)**, 1 mm; **(b)**, 200 µm.

**Supplementary Figure 7. VTA dopaminergic neurons projecting to the nucleus accumbens revealed by cell-type specific tracing.**

**
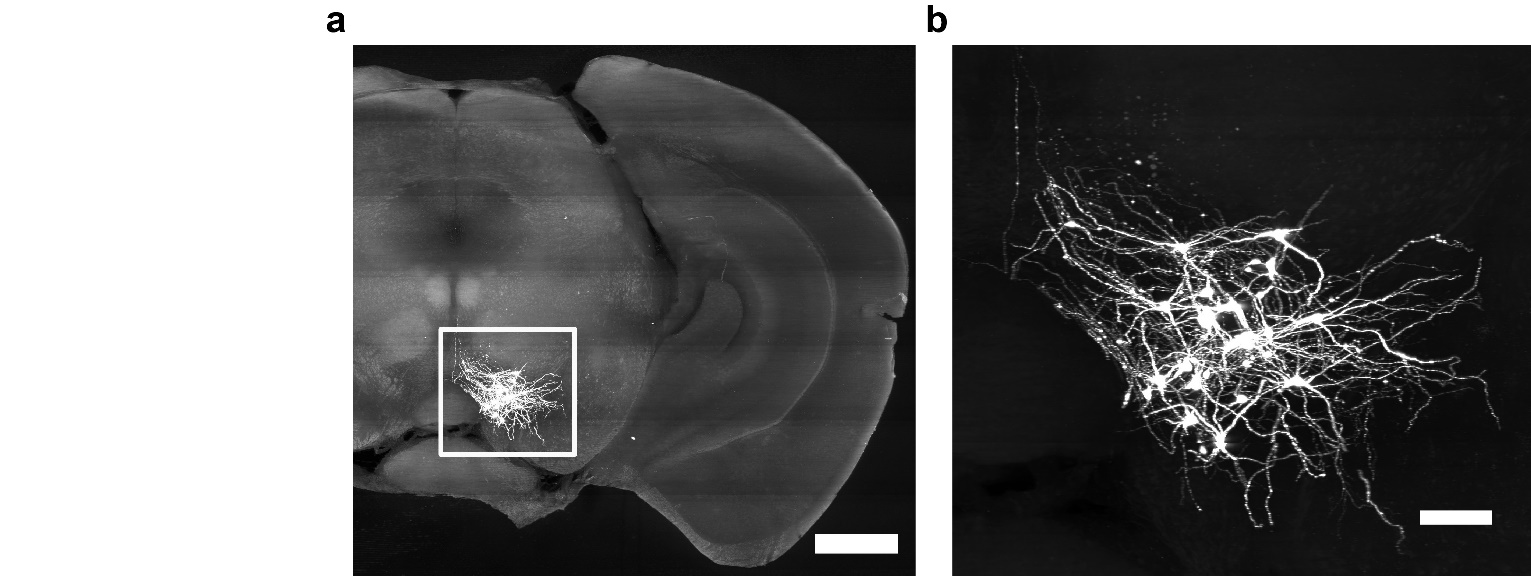
**

**(a)** VTA dopaminergic neurons labeled by rVSV-EGFP-NR7A(A/RG) injected into the NAc in conjunction with rAAV-DIO-BFP-T2A-TVA injected into the VTA of a DAT-Cre mouse. **(b)** The morphology of EGFP-postive neurons clearly resolved by VISoR imaging. Scale bars: **(a)** 1 mm; **(b)** 200 µm.

**Supplementary Figure 8. Cell detecting process.**


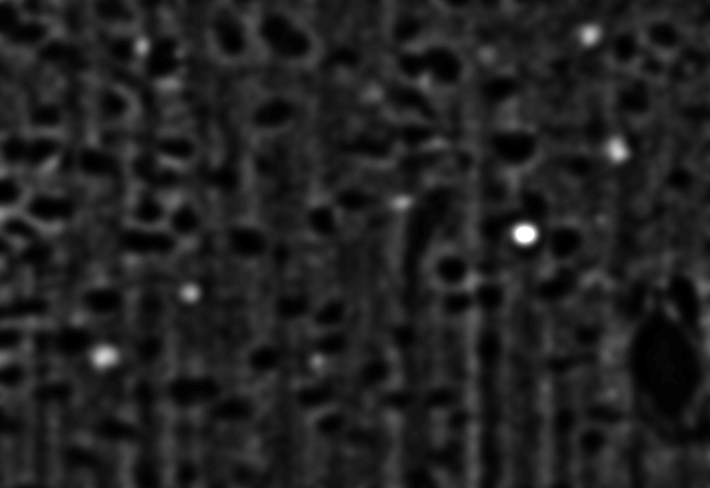


Raw image

Differential of Gaussian (DoG)

Local maxima


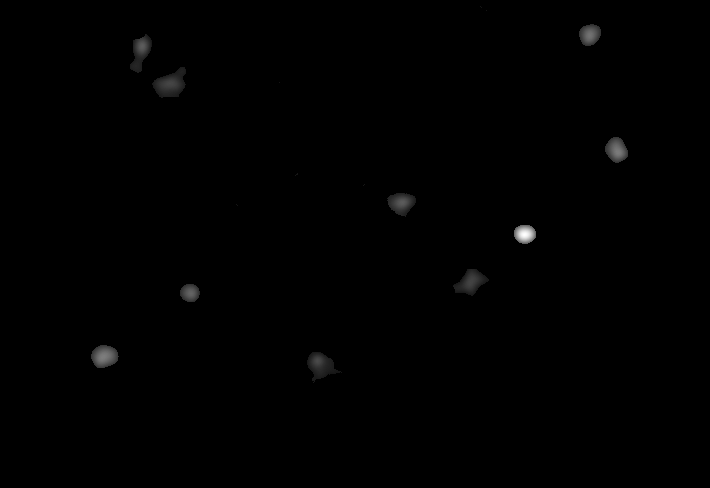


Segment cells


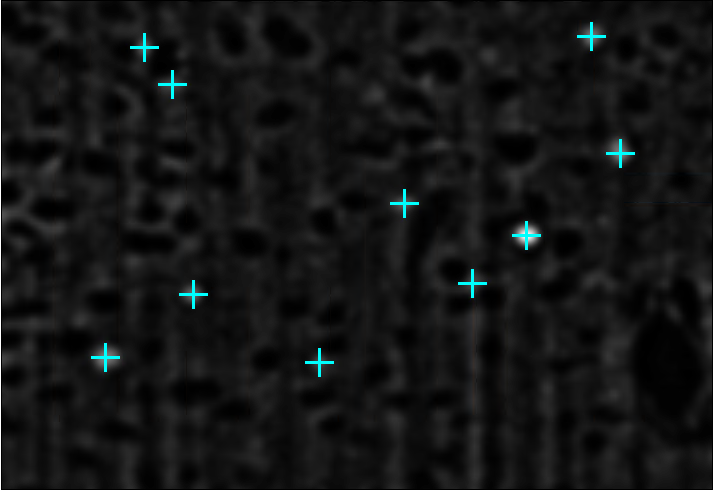

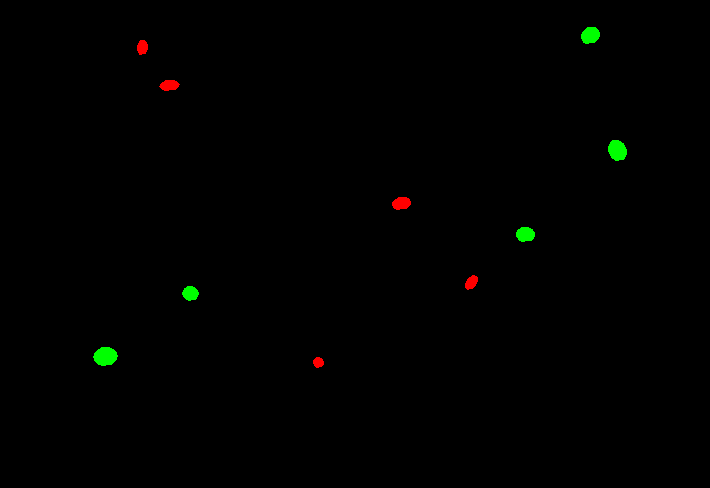


Validation

True

False


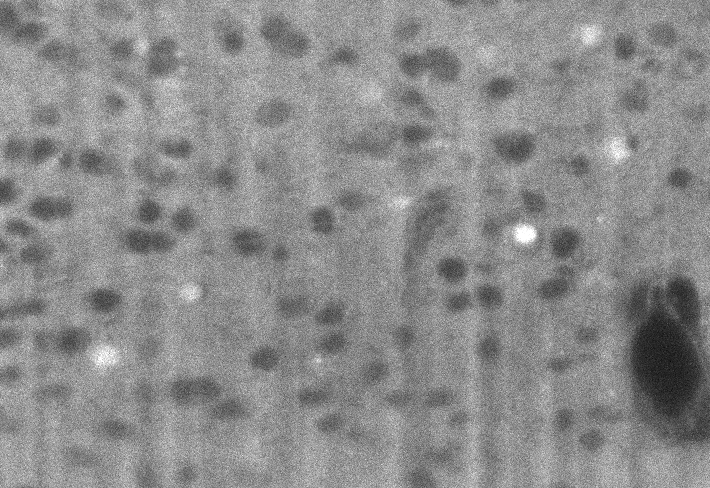


Step I.

Step II.

Step III.

Extract features


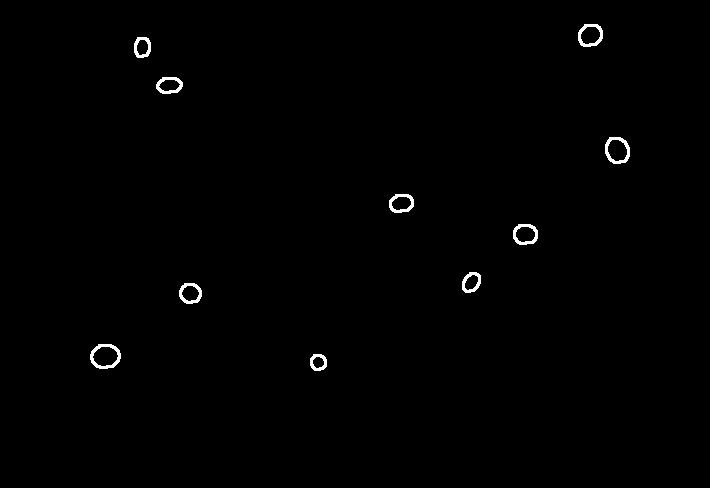

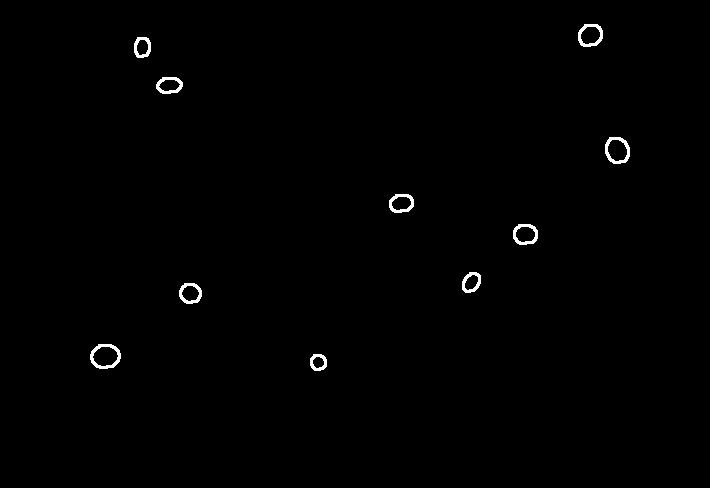

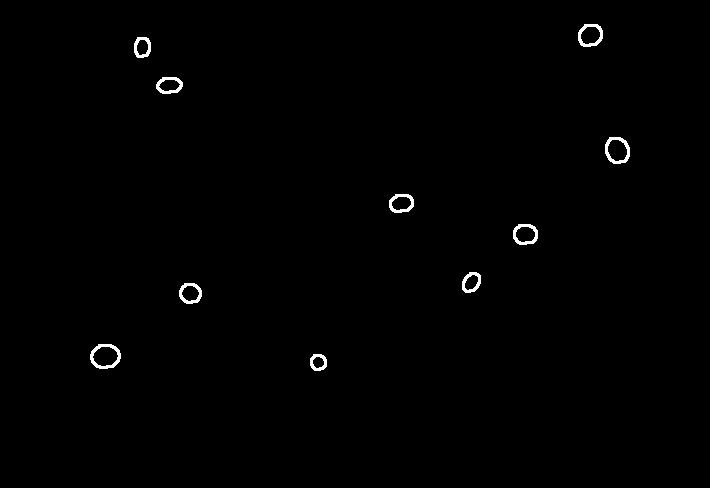


Validation.

Cell detecting algorithm consisted of three steps**. Step I:** Finding all local maxima of DoG (Difference of Gaussian) and dividing them using watershed algorithm. **Step II:** Extracting image patches of surrounding of each local maxima, and find the area of cell by threshold. **Step III.** Aligning areas of cells between neighboring images.

**Validation:** After detection, the position, volume, eccentricity and intensity of cells are calculated, then compared to predefined ranges of cell nucleus to validate the detected cell.

**Supplementary Table 1. Comparison with alternative whole-brain imaging methods.**

| Methods  Property | Serial 2p tomography | FAST | fMOST | WVT | LSTM | VISoR |
| --- | --- | --- | --- | --- | --- | --- |
| Voxel size | 0.45 x 0.45 x 1.33 μm | 0.7 x 0.7 x 5 μm | 1 x 0.8 x 1 μm | 0.32 x 0.32 x 1 μm | 0.65 x 0.65 x 5 μm | 0.5 x 0.5 x 3.5 μm |
| Time consuming for whole mouse brain | ~ 1 week | ~2.4 hrs | ~ 19 days | ~3 days | ~9 hrs | ~1.5 hrs |
| Sample preparation | Whole brain DMSO clearing | FPA fixed brain | Resin embedded | Resin embedded | Whole brain CLARITY clearing | Slice CLARITY clearing |
| Staining | No | No | No | PI staining | Immuno/Chemical | Immuno/Chemical |
| Reference | Economo et al.,2016 | Seiriki et al., 2016 | Gong et al., 2013 | Gong et al., 2016 | Migliori et al., 2018 | This study |

**Supplementary Video 1**

VISoR image series from a 300-µm-thick Thy1-YFP brain slice captured at 100 fps, with smooth stage motion and synchronized scanning illumination. Fine structures of cortical neurons are revealed. Playback is at 0.1X real-time acquisition speed.

**Supplementary Video 2**

VISoR image series from a 300-µm-thick Thy1-YFP brain slice captured at 100 fps (top-left corner) with maximum intensity projections of these images after transformation and alignment. Playback is at the actual acquisition speed.

**Supplementary Video 3**

Navigation through an entire reconstructed Thy1-YFP mouse brain slice showing detailed neuronal structures.

**Supplementary Video 4**

Reconstructed whole brain of a Thy1-YFP mouse imaged by VISoR. The fluorescence intensity of each slice is adjusted according to its local contrast in order to enhance the global visualization clarity.

**Supplementary Video 5**

Whole brain neuronal activation labeled by green dots as single neurons in c-Fos-shEGFP mice brains are compared between control and forced swimming stressed animal.
